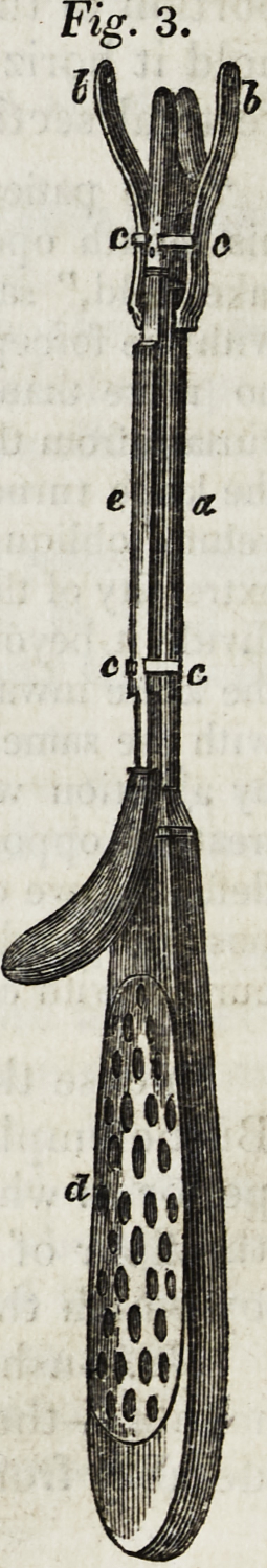# An Essay on the Operation for Cleft Palate

**Published:** 1836-10

**Authors:** 


**Art. IV.**—*An Essay on the Operation for Cleft Palate.* By George Bushe.—*New York*, 1835. Quarto, pp. 20; with Plates.

One of the consequences of the more accurate knowledge we now possess of the manner in which reparation is effected in living bodies, is a diminution in the number of those diseases and malformations which were at one time committed entirely to the skill of the machinist. His contrivances, however skilfully managed, must notwithstanding be considered as the opprobria of surgery; for this science consists in aiding and directing those powers which the body possesses of mending its own defects, and not in repairing it as a mechanic does a machine. Science is therefore much indebted to Dupuytren, Gräfe, Dieffenbach, Roux, and others, for having directed their attention to modes of remedying by operation cleft palate, artificial anus, the loss of the nose, recto-vaginal and recto-vesical fistulæ; and thus enlarging the domain and extending the usefulness of surgery. Our own surgeons have not been behind their continental brethren in their useful operations; and the present essay, by Mr. Bushe, of New York, proves that the same spirit prevails among the most esteemed operating surgeons in America. Mr. Bushe, who is a surgeon of much celebrity, having had occasion repeatedly to operate for cleft palate, has been led to devise plans for its execution, which have proved so effectual that he has been induced to publish them; and, if the report be correct that the late fire at New York destroyed almost the whole of the impression of Mr. B.'s essay, we shall hope to be the means of giving his views a wider circulation than they could otherwise have gained.

As far as it goes, Mr. Bushe's treatise is valuable. It is very clearly and circumstantially written, and, although relating to an operation now familiar to most operating surgeons, it contains several particulars well deserving attention. We should have been still more indebted to Mr. Bushe, if he had presented to us something new on the more difficult subject of fissure of the bony palate; but he takes no formal notice of this branch of the subject. His instruments are both complex and minute; and the remarkable success said to attend the operation in the hands of Mr. Bushe has induced us to incur the expence of drawings of them.

The causes which Mr. Bushe believes to contraindicate the operation for closure of a fissure of the soft palate are the following:

1. Infancy, childhood, and boyhood; as the mouth is too small for the necessary manipulations; resolution and acquiescence are insufficient; and abstinence, particularly from drink, is less easily borne, nutrition being more vigorously carried on.

2. Affections of the lungs attended with cough, or affections of the stomach with vomiting, eructation, &c., from their dislodging the sutures.

3. Diseased tonsils, or other diseases of the fauces, from their creating irritation.

4. Excessive irritability, from its producing continual cough and endeavours to detach mucus from the fauces.

This state of the system may be ascertained by passing instruments into the pharynx, and detaining them for a moderate length of time; when, should the patient be irritable, he becomes anxious and restless; also by studying his character, and enquiring into his conduct in former illness. Although this state depends much upon organization, yet it is often produced, and always increased, by mental and bodily indisposition; therefore the operation should be postponed until, by proper regimen, medicine, and constant exercise of moral restraint over his feelings, the infirmity be removed or palliated.

5. Preternatural thirst is an insurmountable obstacle to success; as the heat, dryness, and constriction of the fauces, as well as the viscidity of the saliva and mucus, will become so intolerable, that the patient cannot resist drinking; and, indeed, a moderate quantity should in such circumstances be allowed.

This may depend on disease, habit, or organization. "If the consequence of disease, appropriate means must be employed to combat it; if of habit, moral discipline should be strictly enjoined; and if of organization, the experiment ought to be made, whether, by repeatedly injecting fluid in small quantities into the rectum and bathing the surface, the thirst can be sufficiently allayed for a length of time equal to that during which we are generally compelled to withhold drink after the operation." Provided then this experiment succeeds, or the disease is removed, or the habit broken, the operation may be undertaken, but not otherwise.

6. Hot weather is obviously disadvantageous.

7. Complete separation of the bony palate to the extent of an inch will render the case unfit; as in such instances each half is directed considerably upwards and inwards, which creates such tension that the sutures are soon torn out of the velum.

When none of these objections exist, the following points should be attended to:

1. For a few weeks previously, the skin and mucous membranes should be brought into a healthy state by exercise in the air, diet, &c.

2. A month before, two thirds of each half of the uvula should be removed; otherwise, after the coaptation of the velum, they will be separated by the pressure of the base of the tongue, and, being swollen, will irritate the fauces.

3. During the previous week, blunt instruments should be passed into the fauces daily, and the jaws should be retained open for as long a period as possible, to accustom the parts to the protracted duty they will have to perform.

4. The operation should be commenced four hours after a meal, in order that digestion may have been performed, lest irritation of the fauces should produce vomiting.

Mr. Bushe divides the operation into two stages,—the section of the palate and the insertion of sutures, which he now performs with an interval of four hours. In this he differs from other surgeons, some of whom have first inserted the sutures and then divided the velum; but Mr. Bushe found that the sutures produced so much irritation and muscular action, that it was difficult to cut the velum evenly, and that the sutures were in danger of being cut, and interfered with the action of the knife. He also found that, when he divided the velum first, and then inserted the sutures immediately, that difficulty arose from the irritation of the muscles and from the bleeding; and consequently he now waits four hours, directing the throat to be gargled with iced water in the interval. This allows the spasm of the muscles to subside, and the parts obtain their ultimate degree of tumefaction, so that the ligatures are not tied too tight nor too slack.

Great care should be taken in cutting the borders of the velum perfectly smooth, and through its whole thickness: from half a line to a line, or even more, should be cut off. For this purpose Mr. Bushe employs a knife and forceps of somewhat peculiar shape. The blade of the knife is one inch and a quarter in length, one-sixth of an inch in breadth at its widest part, and sharp on the back for a quarter of an inch, with a slender handle five inches long. He prefers this to Dieffenbach's double-edged lancet-shaped scalpel, which is apt to cut the velum out of the line of incision with its posterior edge, as it is broader and not blunt on the back. The forceps (Fig. 1,) is seven inches in length, and so bent that the operator's hand may correspond to the side of the patient's face; thus permitting a clear view of the interior of the mouth, an object of great importance not hitherto attained.

*Fig.* 1.

"The extremity of each chop of this forceps is half an inch long, three-eighths broad, and one-sixteenth thick; they stand out at nearly a right angle from the curve, and are furnished, one with a groove, (*a b*,) and the other with a projection adapted to the groove," (*a c*.)

Thus the surgeon can seize a considerable portion of the entire thickness of the velum, and hold it horizontally, so as to ensure a smooth and vertical section.

"The patient being seated near a lofty window, with his mouth open, and his head fixed by an assistant, I take hold," says Mr. Bushe, "of one side of the velum with the forceps, being careful to embrace as much and no more than will prevent the muscles retracting this curtain from the mucous membrane. With the back of the knife turned towards the pharynx, I now transfix the velum obliquely upwards and backwards close to the extremity of the forceps, and, by a steady sawing motion, divide it beyond the angle; then, turning the edge of the knife inwards and downwards, I complete the section with the same sawing motion. In a few minutes, when, by ablution with iced water, the hemorrhage ceases, I treat the opposite side in a similar manner. Should a portion of the bony palate be deficient, we ought to detach a few lines' breadth of the velum on each side from the posterior border of the palate bone, which enables us to approximate the edges of this curtain with comparative ease." (P. 12.)

In case the patient be unsteady and cannot keep his mouth open, Mr. Bushe employs a speculum. It consists of two transverse bars, the superior of which is adapted to the shape of the palate, and the inferior to the floor of the mouth: these are connected by a vertical bar and screw, by which the interval between them is adapted to the size of the mouth.

Mr. Bushe completes the second part of the operation with two instruments,—the suture instrument and the knot-maker; which differ considerably from those hitherto in use, and seem both ingeniously contrived and well adapted for their purpose. We give accurate representations of them, and the author's minute account of the manner of using them.

*Fig.* 2.

"When the second period of the operation arrives, I complete it with the following apparatus, viz.: suture instrument and knot-maker. The suture instrument (Fig. 2,) consists of three parts; the first (*a*) which is inserted into a handle similar to that of an aneurism needle, is four inches and one-quarter in length, one-sixth of an inch broad, and one-eighth thick, except about an inch and three-eighths from its distal extremity, where it is contracted to one-half its size, becomes circular, and is bent, nearly corresponding to the curve of a dissecting hook. Two small rings (*b b*) are attached to it, one being situated on the most convex part, and the other one inch and an eighth nearer the handle. These rings are for transmitting the ligatures, which are thus prevented from becoming entangled or interfering with the sliding bar. The second, or needle, (*d*) is three-eighths long, and consists of three parts, viz.: the blade, which is one-sixth of an inch in length, triangular, and furnished with shoulders; the middle, which is one-eighth of an inch long, and perforated for the transmission of the ligature; and the upper blunt or narrow prolongation, which is one-twelfth of an inch long, and is lodged in the extremity of the curve of the first part. The third and last part of this instrument is a sliding bar (*e*) five inches and five-eighths in length, one-twelfth of an inch thick, and one-sixth broad, terminating at its proximal extremity, in a small handle, (*f*) and furnished at the other, which is round and fiveeighths of an inch in circumference, (*g*) with a spring (*i*) and cavity (*h*), for the reception of the blade and middle portion of the needle. This bar is connected with the body of the instrument by two bands, about an inch apart, and one inch and a half from its distal extremity, which stands outwards, so as to range with the needle.

*Fig.* 3.

"The ligatures should consist of two threads of three-twist silk, and be three feet in length: having passed the extremity of one ligature through the eye of the needle, then carried both extremities through the rings, and twisted them round the handle so as to secure the needle firmly in its socket, the curved extremity of the instrument should be carried behind the velum at the base of the uvula, and then brought forwards through this curtain three or four lines from its border, forcing it from behind forwards and from above downwards. When the point of the needle appears through the velum, the sliding bar should be pushed onwards until it embraces the needle, and, through the medium of the spring-catch, holds it firm while withdrawn. The other extremity of the ligature is to be carried through the opposite side of the velum in the same manner. Another ligature should then be passed between the first and the angle of the wound, which in the majority of cases will be sufficient.

*Fig.* 4.

"The last part of the operation consists in tying the knots; and this I accomplish with the instrument represented in Figs. 3 and 4. This instrument consists of two parts, viz.: one for making the noose, and the other for retaining it in close contact while the second knot is made. The first part, or that for tying the knot, (Fig. 3,) is fixed in a handle like the suture instrument, is three inches and three quarters in length, one-sixth of an inch broad, and one-twelfth thick. It forks about one inch and a quarter from its distal extremity, the ends of the prongs (*b b*) being five-eighths of an inch apart, and bored for the transmission of the ligatures. About three quarters of an inch from the handle on its anterior face, is a ring (*c c*) with the superior portion cut out to the extent of one-tenth of an inch; another (*c c*) arises from the prongs, seven-eighths of an inch from their extremities, for the accommodation of which, they are compressed in this situation. The second portion (Fig. 4,) consists of a slender bar, (*a*) four and a half inches long, furnished at the proximal extremity with a small handle, (*b*) and at the distal with the forceps (*d e f* ). For three-eighths of an inch from the handle, and the same distance from the forceps (*c c*), this bar is square, and one-twelfth of an inch in diameter; between these portions, it is one-eighth of an inch broad, and one-sixteenth deep. The forceps is seven-eighths of an inch long, the inferior portion of which is continuous with the bar just described, but, on a line one-twelfth of an inch below it; while the proximal extremity of the superior portion is furnished with a strong spring (*h*) that works upon the inferior. Both portions are connected by a semilunar joint (*g*), and the chops are three-eighths of an inch long. This second portion of the instrument is placed on the first and secured in the brackets above described, with the chops of the forceps open, because of the pressure of the distal bracket on the superior portion of the forceps.

" Before tying the ligatures, we ought gradually to draw each of them forwards, and, while holding them steadily so as to prevent any drag on the palate, an assistant should wax them anew; a proceeding of the utmost importance, for by their long stay in the mouth they are apt to collapse, and the knots to give way. This being done, the lowermost ligature should be tied first, in the following manner:—the ends being passed through the holes in the prongs of the instrument, the latter should be pushed onwards until the edges of the palate are approximated, taking care not to tie too tight, else the ligatures will destroy the vitality of the parts included; and here we find the advantages of allowing the velum to swell before we pass the sutures, as then we know how tight we ought to tie. The knot being made, the forceps should be pushed forwards, so that it may grasp the knot by virtue of its spring, the counteracting force being removed by change of position. By the greater narrowness of the bar near the forceps and handle, it will now slip out of the brackets and remain hanging from the mouth, while we make another noose, which is to be treated in the same manner. The second ligature ought then to be secured like the first, and thus the operation will be completed." (P. 14.)

The subsequent treatment is most important. The patient should avoid coughing, swallowing, and speaking; sit erect, in order to avoid sleeping; for, when asleep, the throat dries, and the patient is apt to start up and tear out the sutures in an effort to swallow. A cup of beef tea should be injected every two or three hours into the rectum, to sustain the powers and to supply the blood with fluid: if this quantity purges, it should be diminished. The mouth should also be rinced with iced water, to keep it cool and allay thirst. If there is a tendency to cough, five drops of laudanum should be given occasionally. The upper suture should be removed on the fourth, and the inferior on the fifth day, as they then become loose, and are apt to create erysipelatous inflammation and ulceration.

Besides the instruments, the novelties introduced by Mr. Bushe consist in waiting four hours until he inserts the sutures, and in applying only two. For both of these modifications Mr. Bushe gives solid reasons: for the first, the subsidence of spasm and the waiting for tumefaction; and for the second, the fact that he has rarely seen adhesion take place at the points where the sutures were applied. Mr. Bushe's remarks on the causes which contraindicate the operation, and the preparatory and after-treatment, are those of a judicious practical surgeon; whilst the ingenuity displayed in the instruments for making the sutures proves him to be an adept in the mechanical department of his art.

We conclude this notice with the account of a successful operation for cleft palate, performed by Professor Deckmann, and which we extract from a late Number of Kleinert's Repertorium. It will to many readers have a peculiar interest, when it is understood that the narrator and the patient is one and the same person, and a member of our own profession.

"The patient had the misfortune to be born with a divided soft palate, by which malformation he was prevented, when an infant, from taking the breast. He acquired a nasal and indistinct tone of voice, and could not swallow when in a horizontal position, as the food, particularly that of a liquid kind, slipped into the trachea: at length he was unable to expire the air through his mouth, without at the same time closing up his nostrils. He accounted for this malformation by the circumstance that his mother, while pregnant, had been alarmed at seeing a man similarly afflicted exhibit his deformity by yawning.

"The palatine fissure was about one and a half inches in length, at the lower part three-fourths of an inch in breadth. It extended down to the uvula. The patient had undergone an operation in the year 1831: the adequate degree of adhesive inflammation, however, did not take place; the lips of the wound separated during a fit of sneezing two days after the operation, when only a small portion of the upper part had healed. The patient, however, underwent a second operation on the 4th of October. The margins of the fissure were removed with a cataract knife; the lower part of each margin being held with pincers. As soon as the application of cold water had arrested the bleeding, the ligatures were applied. These consisted of silk threads, of which two and two were twisted together, well waxed and afterwards pressed flat with the nail, to prevent their cutting. Three of these were found sufficient to unite the lips of the wound. Their application occasioned retching. The operator made use of Gräfe's needles; short and more curved ones for the upper, but longer and less curved ones for the lower ligature. They were applied with Gräfe's needle-holder, in such a manner as to penetrate from behind towards the front, and were then drawn out with a pair of fine nippers. The upper ligatures were applied first. Great inconvenience ensued from the continued influx of blood into the throat, which, together with an abundant secretion of mucus, occasioned frequent coughing and efforts to clear the throat. The patient having recovered himself a little, the operator proceeded to unite the lips of the wound. The upper ligature was first drawn together in a knot with the fingers, and then retained in its position by an assistant with the aid of pincers, whilst the two succeeding knots were accomplished in the same manner. The lips of the wound were now perfectly drawn together. The patient remained in an upright position, with his head bent a little forwards, and afterwards maintained a similar position in bed, in order that the saliva might flow off the more easily. He was much disturbed by a cough during the night and the succeeding day, but, by great attention, this had no influence on the movement of the palate. Whenever he coughed, he opened his mouth as wide as possible, bent his head forward, formed a hollow in his tongue with the concavity upwards, the point resting against the hard palate so as to press down the root of the tongue as far as possible.

"The inflammatory redness and the pain had somewhat increased; in other respects he felt perfectly well, with the exception of occasional but slight rigors. Nothing remarkable occurred the next day, except that a degree of languor ensued, and that the saliva had a bad smell and taste, probably owing to the total abstinence which the patient observed. On the morning of the fourth day the patient took food for the first time, consisting of the yolk of an egg and a small portion of milk. He now began to experience more hunger and thirst than during the preceding days. The latter sensation was allayed by frequently taking cold water into his mouth. The two upper ligatures were removed on the fifth day, the lower one on the seventh. This latter one had cut deeper than the rest, and had caused a corresponding degree of suppuration. The patient now ventured, with great caution, to speak, and at first only to whisper. He rinced his mouth repeatedly with wine, to strengthen the still relaxed parts which had been operated upon. All distress in speaking and in swallowing at length entirely left him, and his speech became greatly ameliorated."

## Figures and Tables

**Fig. 1. f1:**
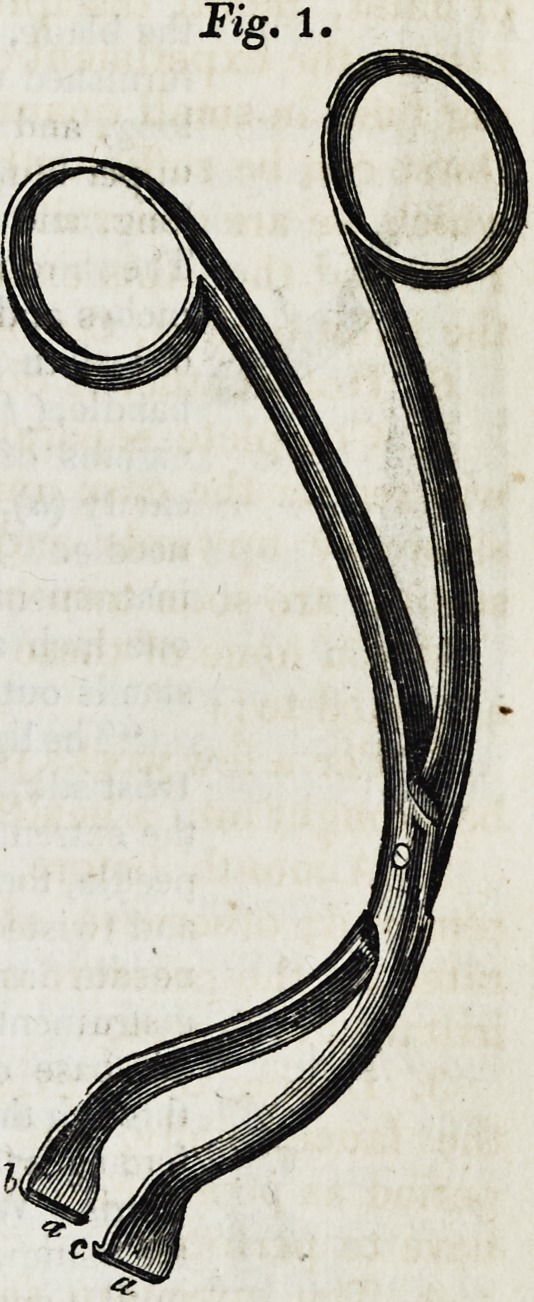


**Fig. 2. f2:**
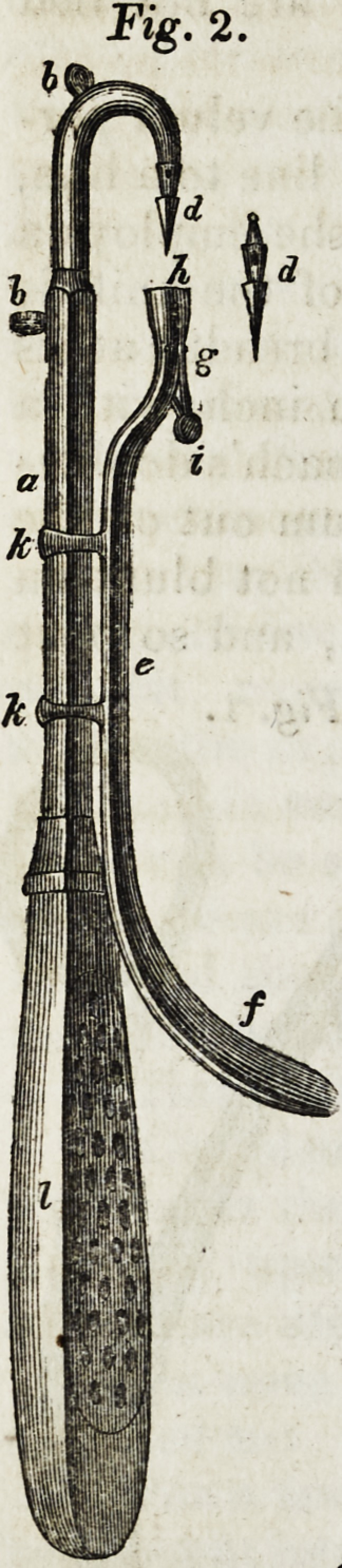


**Fig. 4. f3:**
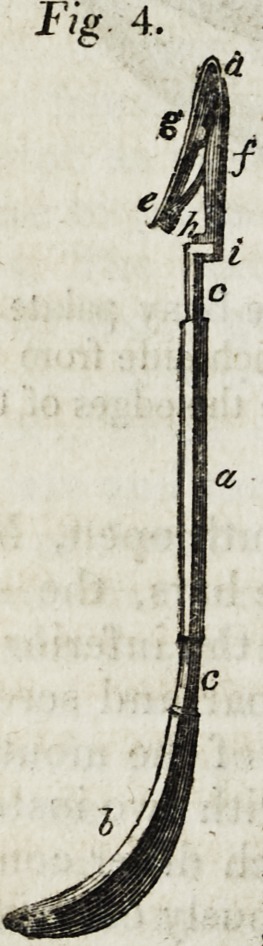


**Fig. 3. f4:**